# Analyzing laboratory test utilization trends in belgian primary care: a decade of insights with international perspectives

**DOI:** 10.1186/s12875-024-02536-9

**Published:** 2024-07-25

**Authors:** Ahmed Medhat Zayed, Nicolas Delvaux

**Affiliations:** 1 Department of Public Health and Primary Care, KU Leuven, Leuven, Belgium; 2 Laboratory Medicine Department, Menoufia University National Liver Institute, Shebin El- Kom, Egypt

**Keywords:** Laboratory Test utilization, Electronic Medical Records Analysis, Primary care in Belgium

## Abstract

**Background:**

Clinical laboratory testing, essential for medical diagnostics, represents a significant part of healthcare activity, influencing around 70% of critical clinical decisions. The automation of laboratory equipment has expanded test menus and increased efficiency to meet the growing demands for clinical testing. However, concerns about misutilization remain prevalent. In Belgium, primary care has seen a dramatic increase in lab test usage, but recent utilization data is lacking.

**Methods:**

We conducted a comprehensive retrospective analysis of laboratory test utilization trends within the primary care settings of Belgium over a ten-year period, spanning from 2012 to 2021, incorporating a vast dataset of 189 million test records for almost 1.5 million persons. This was the first study to integrate the metadata from both the INTEGO & THIN databases, which are derived from the two major electronic medical record (EMR) systems used in primary care in Belgium, providing a comprehensive national perspective. This research provides crucial insights into patient-level patterns, test-level utilization, and offers international perspectives through comparative analysis.

**Results:**

We found a subtle annual increase in the average number of laboratory tests per patient (ranging from approximately 0.5-1%), indicative of a deceleration in growth in laboratory test ordering when compared to previous decades. We also witnessed stability and consistency of the most frequently ordered laboratory tests across diverse patient populations and healthcare contexts over the years.

**Conclusions:**

These findings emphasize the need for continued efforts to optimize test utilization, focusing not only on tackling overutilization but on enhancing the diagnostic relevance of tests ordered. The frequently ordered tests should be prioritized in these initiatives to ensure their continued effectiveness in patient care. By consolidating extensive datasets, employing rigorous statistical analysis, and incorporating international perspectives, this study provides a solid foundation for evidence-based strategies aimed at refining laboratory test utilization practices. These strategies can potentially improve the quality of healthcare delivery while simultaneously addressing cost-effectiveness concerns in healthcare.

**Supplementary Information:**

The online version contains supplementary material available at 10.1186/s12875-024-02536-9.

## Background

Clinical laboratory testing constitutes a fundamental pillar in medical diagnostics, representing the most frequently performed activity within the healthcare sector [1]. Despite laboratory testing accounting for a relatively small percentage of total healthcare expenditure -approximately 2% in the United States and Europe- this seemingly minor figure can have a significant impact on patients due to variations in healthcare funding models and out-of-pocket expenses. Nevertheless, it influences up to 70% of critical clinical decisions, including decisions regarding admissions, discharges, and medication prescriptions [2]. Hence, the implications of laboratory testing on healthcare costs extend far beyond the immediate expenditures, underlining the essential role it plays in guiding consequential downstream activities.

The recent surge in laboratory equipment automation has catalyzed an expansion of laboratory test menus, thereby increasing the throughput of clinical laboratories. This increased efficiency addresses the escalating demand for clinical testing [3]. However, there are concerns about the rates of misutilization of laboratory tests, which have been estimated to be very high with underutilization (while understudied) being more prevalent than overutilization (45% versus 21%) [4]. Therefore, improving laboratory test utilization is a key priority for providing better-quality clinical care and eliminating diagnostic errors while safeguarding the cost-effectiveness of the care provided.

Primary care represents a cornerstone of the Belgian healthcare system, mirroring the situation in many other countries. In Belgium, general practitioners (GPs) are responsible for ordering clinical laboratory tests, which are conducted in clinical laboratories. Most laboratory tests are covered by the national health insurance, minimizing out-of-pocket costs for patients. The Belgian Health Care Knowledge Centre (KCE) reported an escalation in the utilization of most laboratory tests in general practice, with the increase ranged from 50 to 300% between 1995 and 2005, based on data extracted from the documents of the National Institute for Health Care Insurance [5]. However, there is a significant gap in knowledge concerning the utilization of laboratory tests in the Belgian primary care in the last 15 years. This lack of recent data hinders the development of effective strategies for optimizing test utilization in primary care. To address this gap and better understand the current patterns of laboratory test ordering, it is crucial to examine the trends and utilization patterns in the more recent years.

In this study, we aimed to study the laboratory test ordering patterns in the last decade by retrospectively analyzing electronic medical record (EMR) databases. Our examination of these extensive databases provided nationwide insights into patient-level laboratory test ordering patterns within primary care in Belgium. We also identified the most frequently ordered lab tests and their patterns over the years. Furthermore, we compared our research results with various studies that have highlighted laboratory test usage patterns in different countries.

## Methods

### Study population

Our study utilized EMR data ranging from January 1, 2012, to December 31, 2021. The data were extracted from two primary care databases in Belgium: the INTEGO database [6] and the Belgian version of The Health Improvement Network (THIN^®^).

**Fig. 1:**
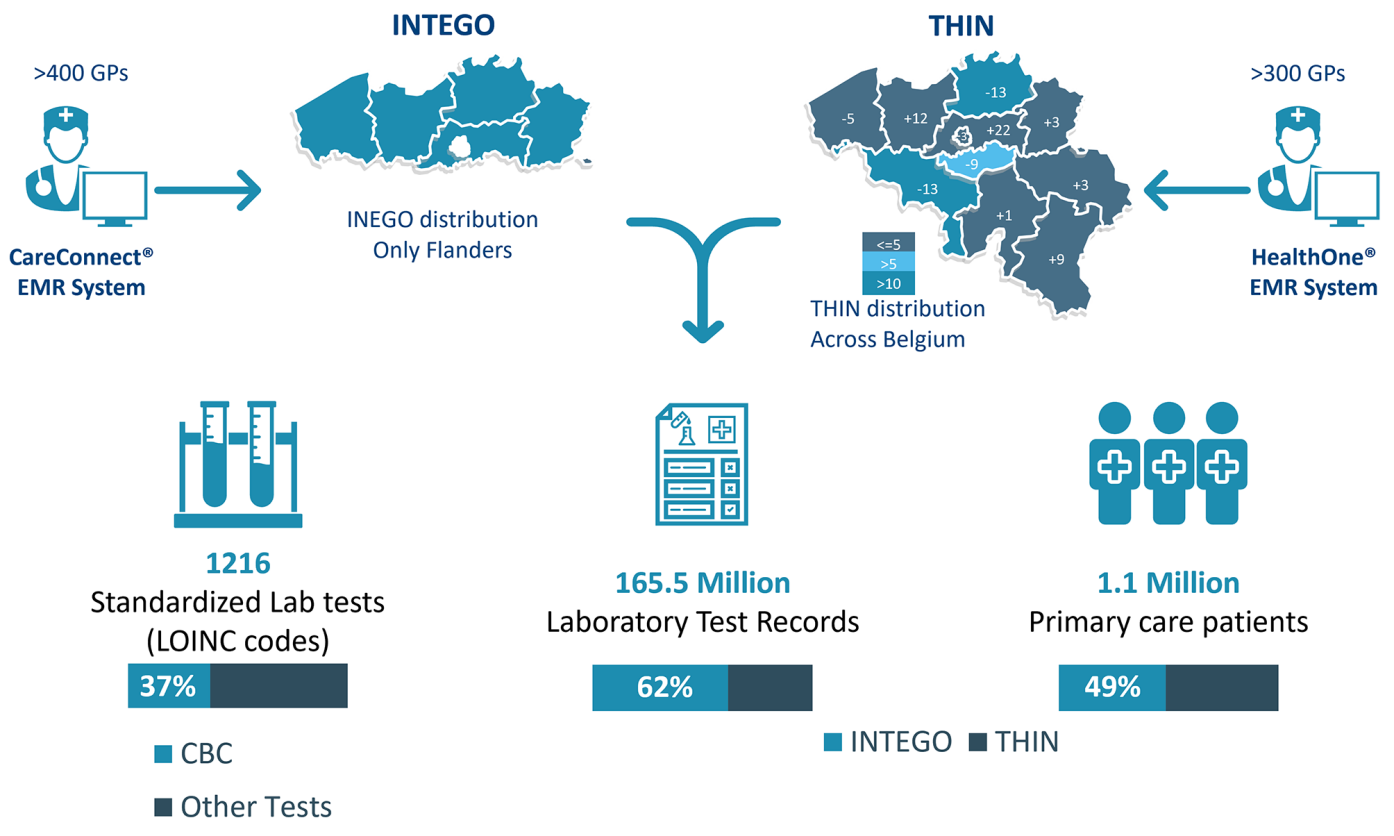
Overview of the two key datasets (INTEGO & THIN) representing primary care in Belgium

The INTEGO database is a Flemish general practice-based morbidity registration network involving over 400 general practitioners (GPs) who use CareConnect^®^ EMR software. This network is coordinated by KU Leuven and covers Flanders comprehensively [6, 7]. THIN^®^ is a private database that is routinely enriched with anonymized medical records provided by more than 300 GPs who use HealthOne^®^ EMR software. The GPs contributing to the THIN database are representative for a majority of Belgian provinces (Fig. 1).

Though they use distinct EMR systems, all participating GPs share their encrypted health records transparently via a third party, which then handles patient-identifiable information before incorporating it into either the INTEGO or THIN databases. As a result, both databases feature clinical data regarding diagnoses, drug prescriptions, laboratory results, and biomedical parameters. As the study was a retrospective analysis using secondary patient data only without reporting on protected health information, the Belgian legislation did not require additional ethical approval and subsequently the requirement for obtaining informed consent was waived.

### Laboratory test data preprocessing

In this study, we extracted laboratory test records from both the INTEGO and THIN databases, each housed in separate tables. These tables solely contained laboratory test data and excluded physical examination findings such as body weight, and vital signs like blood pressure.

Each record in these tables denoted a patient’s result value for a distinct laboratory test identifier on a particular date. The majority of laboratory tests were denoted using local codes allocated by the respective EMR systems of THIN and INTEGO. To guarantee consistency and enable comparison across databases, these local codes were mapped to their corresponding internationally recognized Logical Observation Identifiers Names and Codes (LOINC) through mapping tables. This mapping process resulted in 94% of the test records being matched with 1215 LOINC codes. The remaining records retained ambiguous local codes or test names.

It is worth noting that test panels, such as the Complete Blood Count (CBC), were not recorded as comprehensive panels. Instead, each constituent test within the CBC panel, including Hemoglobin, Leucocyte count, and others, were documented individually. This approach was adopted to preserve granularity and precision in the recording of test results. Given the significant volume of records pertaining to the CBC panel, which constituted approximately 37% of all test records, and the fact that CBC-related tests are seldom analyzed or ordered individually in labs (unlike other panels), we chose to group all constituent tests of the CBC panel together. Thus, if multiple CBC-related tests were ordered for the same patient on the same date, they were considered as a single CBC test for analysis purposes.

During our preprocessing of the data, we systematically evaluated the data from each database. Initially, we computed the annual number of tests for every unique patient in each of the INTEGO and THIN^®^ databases. Subsequently, we calculated the annual number of orders per patient, considering all laboratory tests ordered on the same day as one order. It’s important to highlight here that our analysis was confined to the laboratory data table of each dataset. Consequently, we included only the patients who underwent lab tests (the denominator) among the broader population of patients visiting the GPs.

### Patient-level statistical analysis

We used R (version 4.1.2) for statistical analyses. As a first step to guide the statistical analysis of the patient-level utilization data, we did a preliminary data analysis, which indicated that the annual number of tests per patient in both databases followed a non-normal distribution. The distribution was shown to be highly skewed and has a heavy tail according to the high skewness (INTEGO: 8.3, THIN: 7.2) and kurtosis values (INTEGO: 169, THIN:238).

To gain a deeper understanding of the temporal patterns of test ordering and utilization among patients throughout the study period, a Negative Binomial regression model was implemented. This particular method was chosen due to the count nature of our dependent variable (i.e., the number of tests ordered per year), and the overdispersion observed in the preliminary data analysis, suggesting that the variance (INTEGO: 2053, THIN: 917) was greater than the mean (INTEGO: 35, THIN: 28). The model was specified with the number of tests conducted per year as the dependent variable and the year of ordering the tests as the predictor. A regression approach allowed us to model the count of lab tests as a function of the year, thus quantifying the annual rate of change in test utilization.

Following the Negative Binomial regression analysis, we further sought to investigate potential points of significant change in the trend, particularly during the last two years of the study period (2020 and 2021). The COVID19 pandemic during this time presented an unprecedented external factor that could have influenced the pattern of test ordering and utilization. Joinpoint regression was employed to discern whether there were any significant joinpoints or shifts in the trend during these years. This method allows for the identification of periods where the trend in test utilization might have deviated from its expected trajectory, given the preceding years. By pinpointing such joinpoints, we aimed to provide a more granular understanding of the temporal dynamics of test utilization, especially in the context of the potential impact of the COVID19 pandemic.

### Test-level statistical analysis

To consolidate the laboratory test data and create an annual list of the most frequently ordered tests in primary care in Belgium, we grouped the tests based on their LOINC codes to categorize them effectively. We then merged the test lists from both INTEGO and THIN databases. Furthermore, we calculated the total count of records for each LOINC code over the 10-year period from 2012 to 2021. This analysis enabled us to identify the laboratory tests that were most commonly ordered during the entire duration of the study. To simplify the analysis and gain insights into specific categories of tests, we categorized the LOINC codes into clusters of related tests (such as lipid profile tests).

In our research, we sought to understand the temporal trend in the utilization of each of the top 25 laboratory tests over the 10-year span. For each laboratory test, we calculated the annual rate as the number of tests ordered per 100 patients for each year. We then employed a linear regression analysis, with the year as the independent variable and the rate of tests per 100 patients as the dependent variable. This method allowed us to evaluate the slope of the trend line (i.e., the annual rate of change in test utilization) and to assess the statistical significance of this trend. In this context, the slope estimate provided the average annual change in the number of tests ordered per 100 patients, while a *p*-value less than 0.05 denotes a statistically significant trend. As tests differed in the range of the annual number of tests, we chose to use the number of tests in the baseline year (2012) as the point of reference for calculating the annual average percentage of change instead of the average annual change in the number of tests to give a measure of how much the number of tests per 100 patients has increased relative to where it started. By repeating this process for each of the top 25 tests, we were able to generate a comprehensive picture of how test utilization has evolved over time.

By conducting this comprehensive statistical analysis, we aimed to uncover significant patterns in laboratory test ordering and utilization within primary care settings in Belgium.

## Results

Overall 165,549,401 tests were recorded for 1,117,873 patients over 10 years from 1 January 2012 to 31 December 2021. Both databases, INTEGO and THIN, contributed almost equally to the total number of patients, with THIN accounting for a slightly larger percentage (51%). However, the INTEGO database contained a higher number of test records (62%) (Fig. 1).

In our analysis of the population characteristics from the INTEGO and THIN datasets, we observed generally similar population characteristics. Across the 10 years, both datasets showed a marginal predominance of females, with INTEGO at 56.9% and THIN slightly higher at 57.4%. Notably, the INTEGO dataset captures a somewhat younger demographic, with its average age being 50, four years younger than THIN’s 54. Our study population in both datasets spanned a broad age range from 0 to 101 years old, encompassing both pediatric and adult patients. The differences in population characteristics are relatively small, yet the t-test for age and the chi-square test for gender both confirm that these difference are statistically significant.

### Patient-level trends of laboratory test utilization

**Fig. 2:**
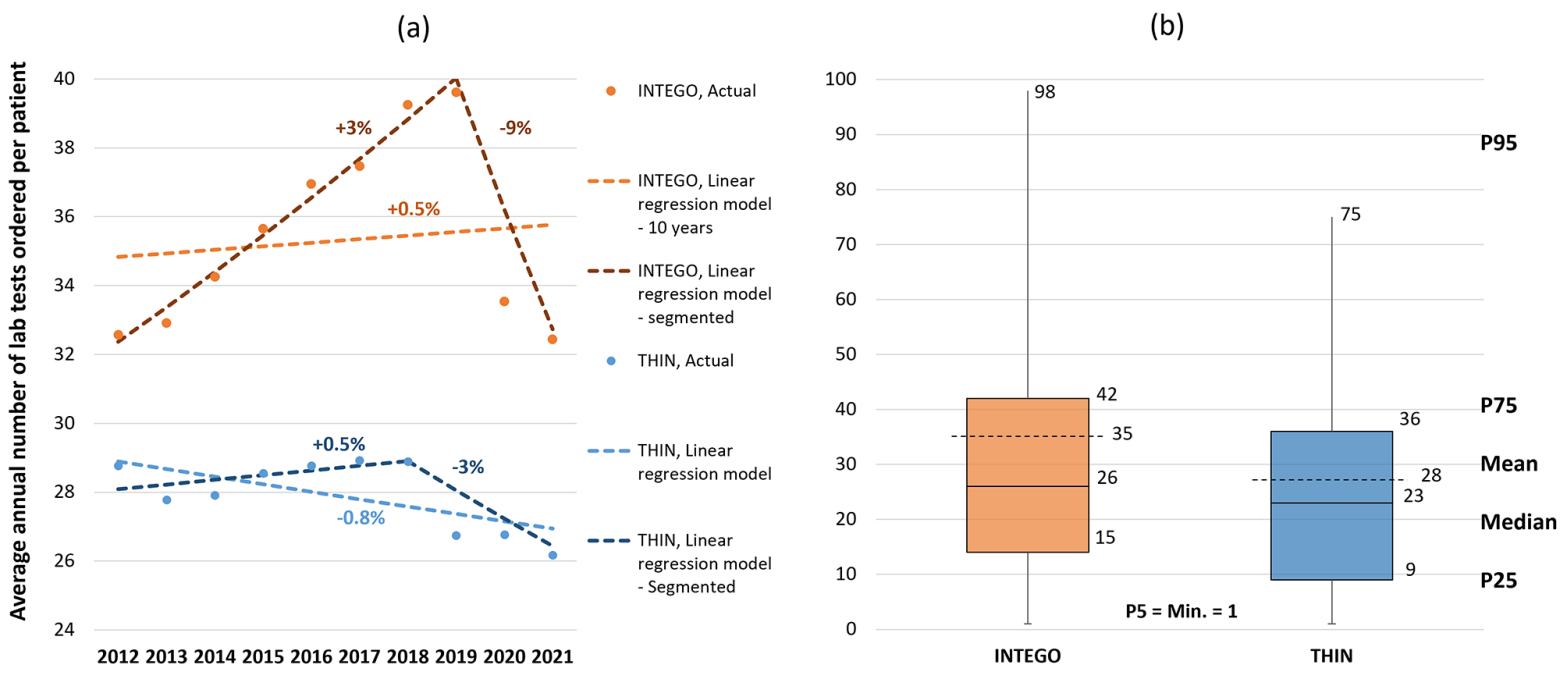
Trends and distribution of patient-level laboratory test utilization: (**a**) Decelerated rising trend of utilization (**b**) Overall distribution of annual lab tests ordered per patient

The Negative Binomial regression analysis (Fig. 2.a) showed that there was a minimal annual increase in the count of lab tests ordered per patient of approximately 0.5% in INTEGO. On the other hand, there was a minimal annual decrease of 0.8% in THIN. Joinpoint regression analysis further clarified the impact of COVID19 on general laboratory test utilization trends. Specifically, there was a 9% downturn in the trend during 2020 and 2021 within the INTEGO database. While the THIN database displayed a similar declining trend of 3%, this trend started earlier to include also the year of 2019 before the COVID19 pandemic. Prior to the COVID19 pandemic, a rising trend in the count of lab tests ordered per patient was noted in INTEGO from 2012 to 2019, with an annual growth rate of 3% and in THIN from 2012 to 2018, with an annual growth rate of 0.5%.

Excluding the COVID19-affected years, both INTEGO and THIN databases exhibited slight yet statistically significant positive trends in laboratory test utilization over the years, with observed growth rates of 3% and 0.5% respectively. However, the INTEGO database consistently documented a higher count of laboratory tests per patient compared to the THIN database as evident in Fig. 2.a. This observation is reinforced by the 10-year distribution displayed in the box plots in Fig. 2.b, where metrics like the 25th percentile, median, mean, 75th percentile, and 95th percentile for INTEGO consistently surpassed those for THIN.

### Test-level trends of the top ordered tests

After grouping all 62 LOINC codes corresponding to the CBC panel, 61.8 million test records (37%) were condensed into 4.9 million CBC records, with an average of 13 test records per CBC order. This approach allowed us to obtain a more accurate representation of the utilization of CBC tests and provided a solid basis for our analysis.

**Fig. 3:**
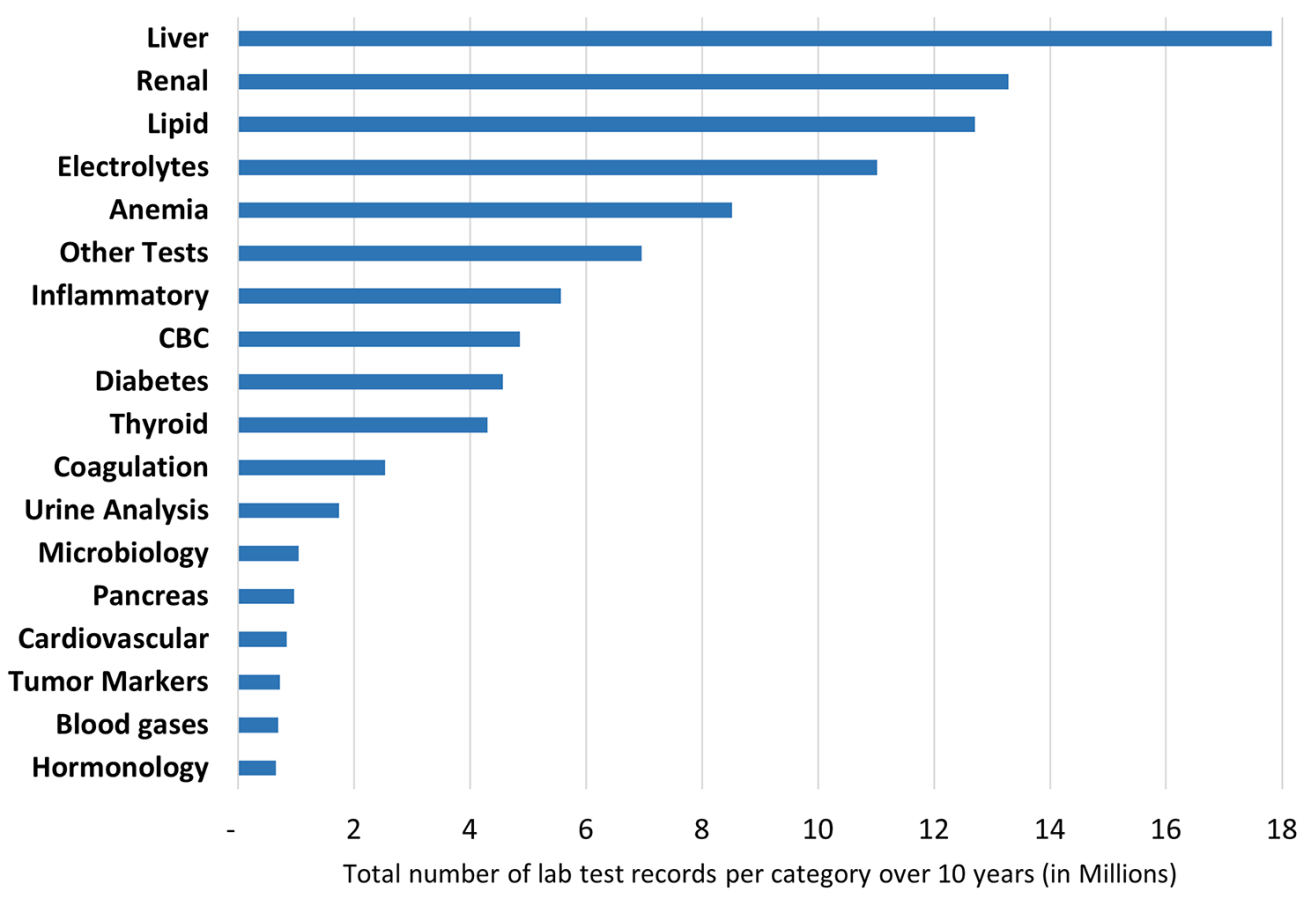
Stable trends and consistency of the top 25 ordered lab tests over 10 years The tests are ranked based on the total number of orders in the 10 years. The test name is given in the abbreviation format, along with the detailed Loinc code. The color of the line indicates the annual percent of change and the type of the line either dashed or not indicates the statistical significance of the trendline

After grouping the CBC test records, our analysis of the top 25 tests ordered over the 10-year study period revealed notable trends in ordering practices (as shown in Fig. 3). Out of these tests, only 11 exhibited statistically significant shifts in average orders per 100 patients. About two-thirds, 7 tests, showed a decline that ranged from minimal (such as LDL Cholesterol with a 0.7% decrease) to moderate (such as Erythrocyte sedimentation rate with a 2.8% decrease). Conversely, four tests, namely Potassium, Glycated hemogloin, Urea, and Folate, showed increasing trends of 1.4%, 1.5%, 2%, and 3.8% respectively. The remaining 14 tests in the list did not show any statistically significant trend. These data, while indicating subtle shifts in laboratory test ordering, underscore a largely consistent pattern in the top 25 test orders over the years.

**Fig. 4:**
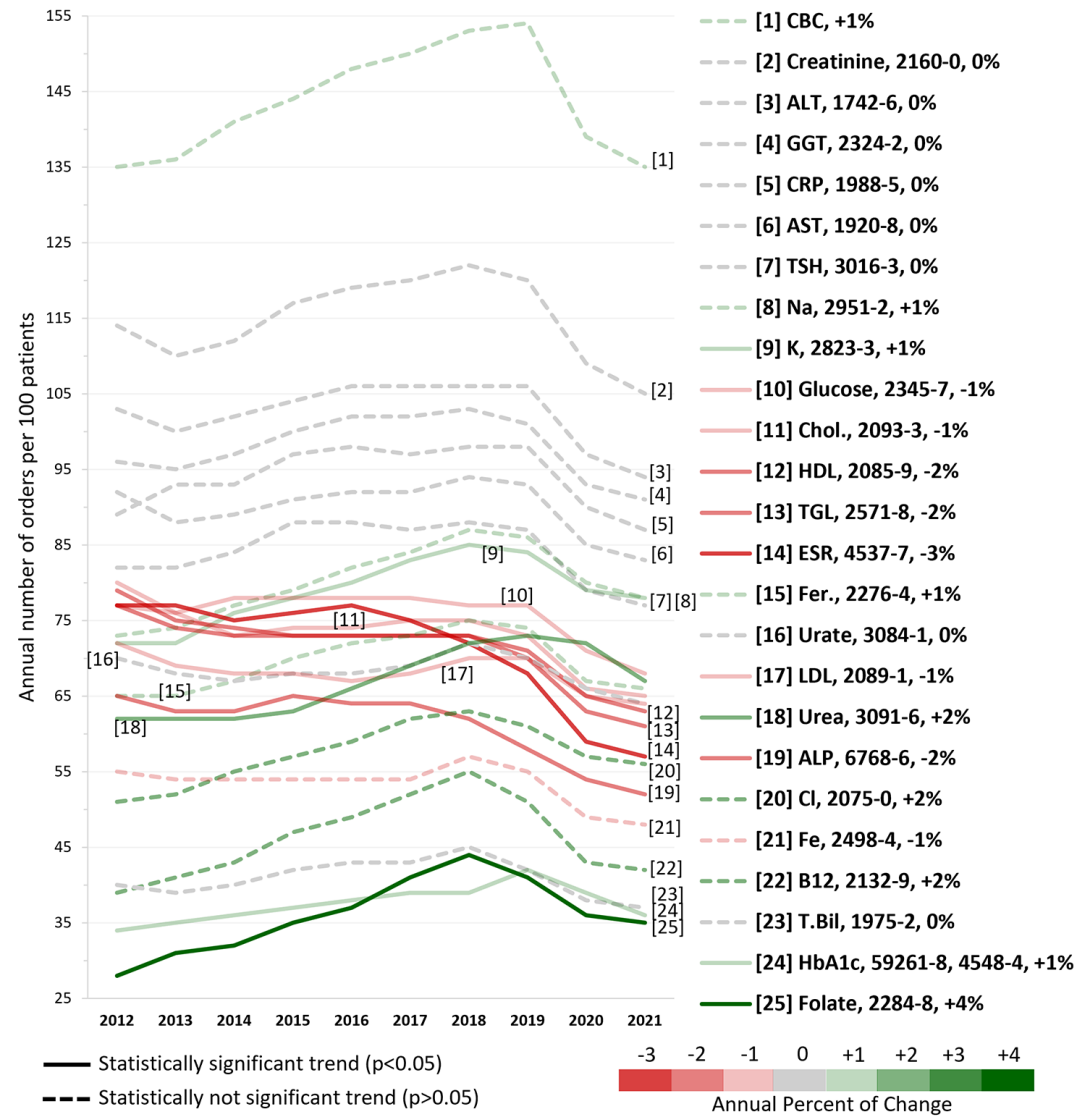
Ranking of top laboratory test groups ordered over a 10-year period

Grouping of the lab tests as shown in Fig. 4 indicates the top ordered groups of tests across the 10 years of study. Liver tests (like Bilirubin and liver enzymes) represented the largest group with nearly 18 million tests. This was followed by renal tests with approximately 13 million, and lipid tests closely behind. Electrolytes and anemia tests also showed significant ordering, with 11 million and 8.5 million tests respectively. On the lower spectrum, tests related to cardiovascular diseases, tumor markers, blood gases, and endocrine tests were ordered less frequently, with each having less than 1 million tests.

### Slowing trends of patient-level laboratory test utilization

Our findings reveal no significant rise in the average annual number of laboratory tests ordered per patient over the past decade, with an annual increase of 0.5% in the INTEGO database and in contrast, a 0.5% decrease in the THIN database. While the KCE report did not provide prior patient-level data from primary care in Belgium for comparison, we can draw parallels with a similar study conducted in primary care in the United Kingdom (UK) [8]. According to O’Sullivan, the annual percentage change in total test utilization per patient decreased from 21% during the period 2000-04 to 7.2% between 2004 and 08, followed by a further decrease to 2.6% from 2008 to 2015. Notably, this observed increase in patient-level test utilization from 2008 to 2015 in the UK closely mirrors the increase we have reported for INTEGO (3%) from 2012 to 2019.

This nationwide minimal annual increase in laboratory test utilization per patient over the last decade may be linked to previously reported rates of laboratory test misutilization, where underutilization rates exceed those of overutilization [4]. However, a more in-depth investigation would be necessary to confirm rates of underutilization. Nevertheless, we should not anticipate a significant surge in overall test utilization. This perspective aligns with our data and its relationship to reported misutilization rates. In light of these findings, we recommend that initiatives aimed at improving laboratory test utilization should prioritize optimizing the utilization. The focus should not solely be on reducing the volume of laboratory test orders. Instead, the emphasis should be on ensuring that test utilization consistently adheres to best medical practices and evolves in response to changing patient requirements. A more nuanced understanding of how health system changes, disease prevalence, and advancements in diagnostic practices may impact laboratory test utilization. For example, the increase in HbA1c testing, driven by the rising prevalence of diabetes, illustrates this point effectively. As healthcare systems evolve to detect and manage conditions earlier and more effectively, laboratory test patterns are expected to reflect these shifts.

### Stability and consistency in frequently ordered laboratory tests

Notably, despite the rapid advancements in diagnostic methodologies, our analysis suggests a stability in the trends of most of the top 25 ordered lab tests in the last decade. These stable trends do not match with the rising trends reported by the KCE for a previous 11-year analysis (1995–2005). In contrast to the increase between 1995 and 2005 (ranging from 50 to 300% for most laboratory tests), there was a minimal annual increase (ranging from 1 to 4%) recorded for only 4 lab tests between the years 2012 and 2021.

Moreover, the list of the top 25 ordered laboratory tests in primary care in Belgium displayed striking parallels to similar lists in published national studies or reports from various countries, including European countries like Denmark (2008–2018) [9], Asian countries such as Taiwan (2015–2020) [10], and even the Medicare data in the United States (summated reports of the years 2014–2021) [11–18]. Interestingly, despite variations in the populations and healthcare contexts of these studies, as well as disparities in the grouping of laboratory tests, all the lists predominantly included the same tests, with only a few exceptions (Supplementary Table 1). We have identified several significant tests that were present in lists from other countries but did not rank within the top 25 in our study. Notably, Serum Albumin, routine urine examination, and Prothrombin time were among the most prominent tests excluded from our top 25, although they did still secure positions within the top 50, which indicates their diagnostic importance. A potential explanation for the infrequent listing of routine urine examinations among the top 25 tests is that these are often conducted at the point of care (PoCT) in general practices in Belgium [19], and such tests may not be consistently recorded in the studied datasets.

The consistent prevalence of the most frequently ordered laboratory tests across various years, diverse populations, and healthcare settings indicates their routine utilization irrespective of the clinical context. Although this observation aligns with the broad diagnostic capabilities of these tests, allowing for the screening, monitoring, and diagnosis of a wide range of medical conditions, it is imperative to prioritize these tests in any initiatives aimed at improving laboratory test utilization. This prioritization should encompass not only optimizing their ordering practices to ensure their orders are not requested solely due to their routine nature but also enhancing their diagnostic yield to ensure that their results are properly interpreted and not overlooked.

### Strengths and limitations

To our knowledge, this is the first study to integrate the metadata from both the INTEGO & THIN databases, which are derived from the two major EMR systems used in primary care in Belgium. By leveraging the two substantial datasets, our study has achieved a feat of offering a broad and comprehensive insight into nationwide patterns of laboratory test utilization with identifying both patient-level and test-level trends. This panoramic national perspective is often a missing piece in many localized studies, positioning our findings as especially significant. Such knowledge can stand as a foundation for developing evidence-based strategies for improving laboratory test utilization. It is important to note, however, that our focus was on traditional laboratory test utilization as captured in EMR databases. This excludes the consideration of Point of Care Testing (PoCT), such as glucose finger prick tests or urine examinations, which are typically not recorded in these databases and are neither extensively utilized nor reimbursed in Belgium.

In consolidating the two databases, it’s crucial to highlight that while there’s a clear delineation with no overlaps between the GPs in THIN and INTEGO, the potential for patient overlaps exists. Nevertheless, given the lack of a unified patient identifier bridging these databases, this potential overlap remains unconfirmed, leading us to set aside this concern in our analysis.

Despite employing uniform preprocessing and analysis methods for both databases, some differences emerged in the examination of the INTEGO and THIN databases. Notably, INTEGO consistently showed higher numbers of annual laboratory tests ordered per patient compared to THIN. This discrepancy can be attributed to the way of in which patient identifiers are anonymized in THIN. Firstly, THIN omits non-numeric result values before migration to prevent potential breaches of protected health information, whereas INTEGO typically includes textual results. Secondly, in THIN, each patient’s identifier is anonymized at the individual GP level, leading to the possibility of a single patient receiving multiple identifiers if seen by different GPs, even within the same practice. Consequently, the total number of lab tests ordered for a unique patient could be dispersed among different GPs. In contrast, INTEGO uses a consistent patient identifier across both GPs and practices.

Additionally, during the COVID19 years, THIN experienced a similar but earlier decline in trends compared to INTEGO, with the downturn beginning a year prior to the pandemic. Notably, the decrease in THIN was less pronounced (-3% versus INTEGO’s -9%). This difference could potentially be attributed to regional variations in the access to laboratory services during the pandemic. This may underscore the importance of considering regional context when interpreting and comparing data across different sources due to geographical factors, including healthcare accessibility, population behaviors, and regional healthcare policies.

## Conclusion

In conclusion, our research bridges a critical gap in recent data, providing insights into both patient-level and test-level trends of laboratory test utilization within primary care settings in Belgium while facilitating international comparisons. Notably, we recorded a minimal annual increase (1–3%) in the average number of laboratory tests ordered per patient over the past decade. As this demonstrates a deceleration in growth compared to previous decades, it underscores the necessity for continuous efforts that aim at optimizing test utilization instead of merely reducing the number of tests but on ensuring their relevance and appropriateness.

Additionally, the stability and consistency of the most frequently ordered laboratory tests, both over time and across diverse populations and healthcare contexts, highlights their diagnostic significance. This suggests that any initiative aimed at enhancing laboratory test utilization should prioritize these tests to maintain their effectiveness and relevance in patient care.

By integrating comprehensive EMR databases, employing rigorous statistical analysis, and considering the broader international healthcare landscape, this study offers a foundation for evidence-based strategies aimed at improving laboratory test utilization, ultimately enhancing the quality and cost-effectiveness of healthcare delivery.

### Electronic supplementary material

Below is the link to the electronic supplementary material.

Supplementary Material 1

## Data Availability

No datasets were generated or analysed during the current study.

## Abbreviations

EMR: Electronic Medical Record
CBC: Complete Blood Count
LOINC: Logical Observation Identifiers Names and Codes
KCE: Belgian Health Care Knowledge Centre
GPs: General Practitioners
