# Supplementary material for: Analyzing laboratory test utilization trends in belgian primary care: a decade of insights with international perspectives
Source: BMC Prim Care. 2024 Jul 25;25:270. doi: 10.1186/s12875-024-02536-9 (PMC11271193; doi:10.1186/s12875-024-02536-9)
Supplement: Supplementary file 1 — Supplementary Material 1 [file 12875_2024_2536_MOESM1_ESM.docx]

| **Country** | **Belgium** | **Denmark** (1) | **Taiwan** (2) | **United States** (3–10) |
| --- | --- | --- | --- | --- |
| Data Source | **Primary Care** | **Central Denmark** | **National Database** | **Reports on Medicare payments** |
| Study Period | **2012-2021** | **2008-2018** | **2015-2020** | **2014-2021** |
| 1 | CBC | Creatinine | Blood Creatinine | Blood test, comprehensive group of blood chemicals |
| 2 | Creatinine | Potassium | Blood Sugar | Complete blood cell count (red blood cells, white blood cells, platelets) and automated differential white blood cell count |
| 3 | ALT | Sodium | Potassium | Blood test, lipids (cholesterol and triglycerides) |
| 4 | GGT | Hemoglobin | Sodium | Blood test, thyroid-stimulating hormone (TSH) |
| 5 | CRP | WBC count | Blood Urea Nitrogen | Blood test, clotting time |
| 6 | AST | ALAT | Whole Blood Count | Hemoglobin A1C level |
| 7 | TSH | C-reactive protein | WBC Differential Count | Blood test, basic group of blood chemicals |
| 8 | Na | Platelet count | GPT (ALT) | Vitamin D3 level |
| 9 | K | Albumin | GOT(AST) | Bacterial colony count, urine |
| 10 | Glucose | Alkaline phosphatase | Triglycerides | Thyroxine (thyroid chemical) measurement |
| 11 | Chol. | Hemoglobin A1C level | Total Cholesterol | Complete blood cell count (red cells, white blood cell, platelets), automated test |
| 12 | HDL | Hematocrit, EVF | LDL Cholesterol | Cyanocobalamin (vitamin B-12) level |
| 13 | TGL | TSH | HDL Cholesterol | PSA (prostate specific antigen) measurement |
| 14 | ESR | Total bilirubin | Hemoglobin A1C level | Ferritin (blood protein) level |
| 15 | Fer. | Urea | Urine Routine | Drug test(s), presumptive, any number of drug classes, per date of service |
| 16 | Urate | Total cholesterol | Blood Uric Acid | Folic acid level |
| 17 | LDL | MCV | Prothrombin Time | Parathormone (parathyroid hormone) level |
| 18 | Urea | Triglycerides | Urine Biochemistry Examination | Natriuretic peptide (heart and blood vessel protein) level |
| 19 | ALP | HDL cholesterol | Blood Gas Analysis | Opiates (drug) measurement |
| 20 | Cl | RDW | - | Detection test for organism |

**Supplementary Table 1: International comparison between the commonly ordered laboratory tests in our study and other studies in different countries for different (sub)populations during different study periods. Studies used different grouping of lab tests. Tests highlighted with gray did not rank among the top 20 tests in our study.**

1. Arendt JFH, Hansen AT, Ladefoged SA, Sørensen HT, Pedersen L, Adelborg K. Existing Data Sources in Clinical Epidemiology: Laboratory Information System Databases in Denmark. Clinical Epidemiology. 2020 Dec 31;12:469–75.

2. Lee PC, Kao FY, Liang FW, Lee YC, Li ST, Lu TH. Existing Data Sources in Clinical Epidemiology: The Taiwan National Health Insurance Laboratory Databases. Clinical Epidemiology. 2021 Dec 31;13:175–81.

3. Medicare Payments for Clinical Laboratory Tests in 2014: Baseline Data [Internet]. 2015 Sep [cited 2023 Sep 6]. Report No.: OEI‐09‐15‐00210. Available from: https://oig.hhs.gov/oei/reports/oei-09-15-00210.asp

4. Medicare Payments for Clinical Diagnostic Laboratory Tests in 2015: Year 2 of Baseline Data [Internet]. 2016 Sep [cited 2023 Sep 6]. Report No.: OEI-09-16-00040. Available from: https://oig.hhs.gov/oei/reports/oei-09-16-00040.asp

5. Medicare Payments for Clinical Diagnostic Laboratory Tests in 2016: Year 3 of Baseline Data [Internet]. 2017 Sep [cited 2023 Sep 6]. Report No.: OEI-09-17-00140. Available from: https://oig.hhs.gov/oei/reports/OEI-09-17-00140.asp

6. Medicare Payments for Clinical Diagnostic Laboratory Tests in 2017: Year 4 of Baseline Data Report [Internet]. 2018 Sep [cited 2023 Sep 6]. Report No.: OEI-09-18-00410. Available from: https://oig.hhs.gov/oei/reports/OEI-09-18-00410.asp

7. Medicare Laboratory Test Expenditures Increased in 2018, Despite New Rate Reductions [Internet]. 2020 Aug [cited 2023 Sep 6]. Report No.: OEI-09-19-00100. Available from: https://oig.hhs.gov/oei/reports/OEI-09-19-00100.asp

8. Despite Savings on Many Lab Tests in 2019, Total Medicare Spending Increased Slightly Because of Increased Utilization for Certain High-Priced Tests [Internet]. 2020 Dec [cited 2023 Sep 6]. Report No.: OEI-09-20-00450. Available from: https://oig.hhs.gov/oei/reports/OEI-09-20-00450.asp

9. COVID-19 Tests Drove an Increase in Total Medicare Part B Spending on Lab Tests in 2020, While Use of Non-COVID-19 Tests Decreased Significantly [Internet]. 2021 Dec [cited 2023 Sep 6]. Report No.: OEI-09-21-00240. Available from: https://oig.hhs.gov/oei/reports/OEI-09-21-00240.asp

10. Medicare Part B Spending on Lab Tests Increased in 2021, Driven By Higher Volume of COVID-19 Tests, Genetic Tests, and Chemistry Tests [Internet]. 2022 Dec [cited 2023 Sep 6]. Report No.: OEI-09-22-00400. Available from: https://oig.hhs.gov/oei/reports/OEI-09-22-00400.asp
